# BK virus and carcinoma of the prostate, kidney and bladder

**DOI:** 10.1038/sj.bjc.6603124

**Published:** 2006-06-13

**Authors:** D B Weinreb

**Affiliations:** 1 Mount Sinai School of Medicine, 50 East 98th Street, Room 11C1, New York, 10029 NY, USA

**Sir**,

We read the recent study by Newton *et al* (2005) with great interest. Presently, we offer a few thoughts on what their results suggest about the role of BK virus in cancer.

First, haemorrhagic cystitis and BK nephropathy in transplant patients are the principal disease entities for which BK virus has been implicated (Hirsch, 2005). We ask, how do the antibody titers of the cancer patients and controls compare to transplant patients diagnosed with BK nephropathy or haemorrhagic cystitis? It would be informative to know whether antibody titers in patients with active BK nephropathy are within the range reported for patients with carcinoma.

Similarly, how do antibody titers in BK nephropathy patients change with treatment and resolution of BK infection? Perhaps antibody titers decline following resolution of the active BK infection to levels comparable to those reported by Newton *et al* (2005). Consider a cancer patient with a history of prior BK infection whose antibody titers were previously high, but now have declined. In such an instance, BK virus may be implicated in carcinogenesis, although the antibody titers at the time of this study have returned to the range of normal individuals. Certainly, a study documenting how antibody titers in patients with BK nephropathy change over time would be enlightening.

Infection with polyomavirus (PV) may both disrupt the function of tumour-suppressor proteins p53 and pRb (Reich and Levine, 1982; DeCaprio *et al*, 1988; Bollag *et al*, 1989; Dyson *et al*, 1990; Harris *et al*, 1998; Pipas and Levine, 2001). As the PV infection is cleared, the ability of the cell to respond appropriately to DNA damage may remain impaired. The cells are then transformed, although the active phase of infection has resolved. We suggest, based on this ‘hit-and-run’ mechanism, that the absence of elevated titers does not exclude a role for BK virus in carcinogenesis.

Nonetheless, the ‘hit-and-run’ mechanism is difficult to defend experimentally. How does one find evidence for an infection that has resolved? We anticipate that this may become an area of active research in the near future.

Finally, Weinreb *et al* (2006) identified a population of patients with PV-infected ‘decoy’ cells seen on urine cytologic analysis. The incidence of bladder carcinoma in this population was significantly higher than in patients receiving urine cytologic analysis but lacking any such infected cells. Their data suggests an association of PV infection with bladder carcinoma. Did any patients in Dr Newton's study have urine cytologic analyses revealing such ‘decoy’ cells? Is there any correlation between the presence of such virally infected cells and antibody titers? The detection of ‘decoy’ cells may be more closely associated with carcinoma than antibody titers.
